# Some Obstetrical Cases

**Published:** 1896-09

**Authors:** W. M. Holladay

**Affiliations:** Hampden Sidney, Va.; Member Virginia Medical Society


### Southern Medical Record.

A MONTHLY JOURNAL OF MEDICINE AND SURGERY.

VOL. XXVI. ATLANTA, GA., SEPTEMBER, 1896. No. 9.

### Original Articles.

### SOME OBSTETRICAL CASES.

BY W. M. HOLLADAY, A. B , M. D., HAMPDEN SIDNEY, VA.
Member Virginia Medical Society.

Some of these cases are interesting to the statistical obstetrician; some as showing result of treatment where several different modes of treatment are advocated—and some as showing the difficulties that beset a country practitioner, where no aid can be found. These, and the expedients that have been found of avail, are more especially for the practitioners so situated. It is well to remark here that the negro race have larger vulvæ than the white; that the axis of the parturient canal is straighter; that its outlet is situated more posteriorly, the line from the posterior commissure of the vulva to the anus being shorter, both absolutely, and relatively to the two lines forming the perineal body, than that in their white sisters; and this must be remembered when making traction with the forceps. Labor, as a rule, is easier in the blacks than the whites, because this straightening of the parturient canal also shortens it.

Four cases of puerperal convulsions of eight treated present points of interest. Of the eight cases four children and eight mothers were saved.

CASE I.—M. B., primipara. Mulatto, *æt.* 17. Convulsions occurred before labor, but were controlled by chloral; when labor occurred the spasms redoubled both in force and frequency. Chloroform was administered, but I have never found it of use in these cases. Morphia was given hypodermically—and shortly after the second injection a well-marked case of poisoning was developed. Total amount given, ½ grain sulphate morphia. When danger from this source was over, blood to the amount of twenty ounces was taken, and some hours later four ounces more. By this time the os was dilated, the woman delivered. There is hardly a doubt that cases of eclampsia treated with morphia in the manner of Veit have succumbed to the treatment—some of the cases as reported showing well-marked symptoms of opium poisoning.

CASE II.—Mrs. N. T., primipara. Convulsions and labor started together. A rectal injection of chloral and a hypodermic of morphia not controlling the spasms, she was bled sixteen ounces. She took chloroform badly, but was delivered with great difficulty, the bones of the dead infant's head being so soft that the forceps would hardly hold. I attribute the trouble with the anæsthetic in these cases to the previous venesection. Where blood-letting has been used in future I would use ether.

CASE III.—Mrs. T., primipara, *æt.* 30. I was called to see her by Dr. J. D. Eggleston of this place. The woman was in a most perilous position, pulse over 130, small and wiry, breathing very bad; os firmly closed; convulsions frequent. She had been bled by the doctor before I arrived, and we shortly bled her again, taking rather more than a pint; the pulse came down to 84, breathing became easy, and convulsions ceased. Labor was terminated by forceps, and after great difficulty the placenta, that had undergone calcareous degeneration, was peeled off. The child a was large, healthy girl. With a quick, small pulse, bleeding looked a dangerous experiment, but the end seemed to justify it. A recent writer to the *New York Medical Record*, advocating veratrum viride in puerperal eclampsia, recommends the advocates of venesection not to generalize from a small number of cases. He reports five with one death. Venesection in my hands has given better results with a larger number of cases.

Dewees in his "Midwifery" calls eclampsia a "ferocious disease," and bleeds largely—96 ounces in one case with recovery.

It is remarkable that the earlier writers report frequently in autopsies, where death was due to puerperal eclampsia, finding blood in the ventricles of the brain. Some of them are Dewees, Dunham, Madame La Chapee. In none of the moderns can mention of blood in ventricle be found. They speak of kidney degeneration, pressure effects; some few of a fatty degeneration of the liver, the etiology being as much a mystery as ever, despite the bacillary theory of Blanc.

One case of eclampsia that I went to see and that was terminated before my arrival, was very remarkable. The husband came to me in haste to see his wife, who, besides having convulsions, was flooding freely. Riding rapidly to his house, we found mother and child sleeping nicely. The woman had flooded profusely—then, says the midwife, the afterbirth came away, immediately followed by the child. Here was a case of placenta prævia, convulsions, and precipitate labor.

Some two years ago I collected all of the cases of Cæsarean section for eclampsia that I could find, and was amazed, out of eighteen cases collected, to find nine recoveries—a percentage of deaths no greater than the best Cæsarean section then gave. I have no doubt that the curative effect in Dhuressen's diagonal cervical incision is due to the blood lost in the operation.

Shoulder cases in the country where one has to administer his own anæsthetic, and has no other assistance than that of ignorant midwives, are the reverse of pleasant. Some years ago I had to deliver a woman who had been in labor two days—the shoulder well jammed down. An old blind woman was my only assistant. Getting one foot down and making traction, I could not get the head to ascend; then failing to get the other foot, I grasped the child's pelvis and rotated the infant on its short axis so as to bring the back forward, thinking to get the other foot. To my great joy, the other leg was brought around, the head in the meantime easily ascending. Since then I have, in shoulder cases, when the belly of the child was anteriorly placed, before finding either foot, sought for the infantile pelvis, and by gentle traction and rotating it, had no difficulty in making the back descend, and grasping the legs—the pelvis is more easily recognized than a leg—the child is more readily turned; and if preferable one can leave the case a breech or half breech presentation. Where it is advisable to deliver quickly, it has the advantage of converting a transverse into a first breech presentation, and of not leaving the rotating to be done in the canal. I have turned and delivered in this mode in less than two minutes.

Mrs. J. J., multipara, white, *æt.* 19, wishes to know why her labors are painless. When in labor she has no backache, nor any other pain; is conscious of the motion of the child, and of interrupted effort. Her labors have lasted about 20 minutes.

N. T., *æt.* 16, was in labor two hours—painless almost entirely. Her case was remarkable in that not enough blood was lost to stain the sheet. There was no tear even of the fourchette. These two seem to be delivering like nature intended them to do. Mrs. J. disliked having so painless a labor. She "did not want to be peculiar."

Mrs. D., multipara, always has a precipitate labor, and it is almost painless. One could easily be in the room with her and not discover that she was in labor. After delivery she is generally in a state of collapse; with great difficulty can the uterus be made to contract, and sometimes she bleeds dangerously. Ten grains of quinine given just before the termination of labor causes the uterus to contract better than any other means. I would prefer delivering any woman I know to Mrs. D., who is a large, fleshy woman with organic heart trouble. Quinine seems to me to be the best of all drugs to stimulate uterine contraction. Fluid extract of ipecac is also very helpful, both to facilitate dilatations of the os and to stimulate uterine contraction. Frequently women in labor are urged by unwise friends to eat too much. In such cases an emetic dose of the fluid extract of ipecac is especially useful. Sometimes, too, the presence of hardened feces in the rectum delays labor, both mechanically and reflexly, and one cannot be too careful to see that the rectum is unloaded.

C. T., primipara, negress, *æt.* 20, when in first stage of labor screamed with pain, and the uterine contractions seemed to have very little effect. Rupturing the membranes, the pains became far more bearable and expulsive. So it seems that in some cases the fluid wedge retards the progress of labor and should be removed, although in the majority of cases it should be retained.

M. J. B., multipara, negress, *æt.* 35, was delivered of a large male child that had neither anterior nor posterior fontanelles. The head of this infant was very hard and could in nowise be moulded by the passage through which it passed. It seemed to be an impossible thing for it to be delivered, but by a most stupendous and magnificent display of uterine and abdominal muscular contraction, it was forced into the world. The fifth day after birth the bones opened and the soft spaces showed. I have never seen a similar case reported.

E. W., primipara, *æt.* 18, negress, slightly contracted pelvis, and after being in labor sixty hours, sent for aid. Infant dead and embryotomy was necessary to remove it. From long pressure on the anterior lip of the cervix, on the fourth day a vesico-cervico-vaginal fistula appeared. An antiseptic injection was given twice daily—Seiler's antiseptic nasal tablets being used—and in less than a month the fistula had healed. The recuperative power of the negro genitals is wonderful. Some years ago, in a case of rupture of the perineum to the internal sphincter, I tied the knees of the woman together, put in a catheter, and had as good a result as if I had operated. Since then I have not infrequently tied the knees together where there was a considerable tear, and there was some cause to delay operation.

In all labor cases in this portion of Virginia, the use of an antiseptic pad over the genitalia is demanded. The attendent nurse is almost always a very superstitious and ignorant negress, whose ideas of cleanliness have not yet been evoluted, and the only manner in which one can get the hands that touch his patient in a moderate way cleansed, is to order an antiseptic wash for the pudenda and an antiseptic pad. The absence of puerperal septicæmia among the blacks is wonderful, as these old women make frequent digital examinations, almost always wiping their fingers on a by no means clean apron if a towel is not handy.

January 1st, 1894, I was called to see a negress whose ninth labor had just terminated. When I arrived I found her in collapse. Making an examination, found the placenta lying in the abdominal cavity and the uterus ruptured from the posterior cervico-vaginal juncture to the fundus. By the time the examination was completed the woman was dead. How that ruptured uterus emptied itself of the child is a mystery. It was impossible to discover if the woman had had fibroid tumor.

